# Neurobiological Abnormalities in the First Few Years of Life in Individuals Later Diagnosed with Autism Spectrum Disorder: A Review of Recent Data

**DOI:** 10.1155/2014/210780

**Published:** 2014-02-09

**Authors:** C. S. Allely, C. Gillberg, P. Wilson

**Affiliations:** ^1^Institute of Health and Wellbeing, University of Glasgow, Caledonia House, Royal Hospital for Sick Children, Yorkhill, Glasgow G3 8SJ, UK; ^2^Centre for Rural Health, University of Aberdeen, The Centre for Health Science, Old Perth Road, Inverness IV2 3JH, UK

## Abstract

*Background*. Despite the widely-held understanding that the biological changes that lead to autism usually occur during prenatal life, there has been relatively little research into the functional development of the brain during early infancy in individuals later diagnosed with autism spectrum disorder (ASD). *Objective*. This review explores the studies over the last three years which have investigated differences in various brain regions in individuals with ASD or who later go on to receive a diagnosis of ASD. *Methods*. We used PRISMA guidelines and selected published articles reporting any neurological abnormalities in very early childhood in individuals with or later diagnosed with ASD. *Results*. Various brain regions are discussed including the amygdala, cerebellum, frontal cortex, and lateralised abnormalities of the temporal cortex during language processing. This review discusses studies investigating head circumference, electrophysiological markers, and interhemispheric synchronisation. All of the recent findings from the beginning of 2009 across these different aspects of defining neurological abnormalities are discussed in light of earlier findings. *Conclusions*. The studies across these different areas reveal the existence of atypicalities in the first year of life, well before ASD is reliably diagnosed. Cross-disciplinary approaches are essential to elucidate the pathophysiological sequence of events that lead to ASD.

## 1. Introduction 

### 1.1. Clinical Importance of Early Identification

Autism spectrum disorder (ASD) is a relatively common, neurodevelopmental disorder with onset of symptoms in the first few years of life. ASD is characterised by difficulties in social communication and repetitive or restricted interests and behaviours [1]. ASDs have high heritability and an unclear aetiology in many cases [2]. ASD is diagnosed in around 1% of the population [3, 4] and was once considered to be a rare psychological disorder due to poor parenting [5]. Despite recent advances in the ability to identify ASD earlier, diagnosis is frequently not made prior to approximately three years. Currently, no reliable predictors of ASD in infancy exist but characteristic behaviours emerge during the second year which are used to aid diagnosis [6–8]. Study into the neurological basis of ASD before the age of three years is imperative [9, 10]. Reliable early identification of neurodevelopmental disorders in childhood in primary care is important as it may improve outcomes [11, 12]. Absence of robust biological markers for identifying ASD has led researchers to focus on behavioural anomalies in order to detect early symptoms of ASD [13]. A number of novel lines of investigation have been used to this end, including retrospective coding of home videos [14–16], prospective population screening [17–19], and “high risk” sibling studies [20–22] as well as the investigation of pre- and perinatal brain development and other biological factors. Early social abnormalities are not easily identifiable in the first year of life in infants who later receive a diagnosis, since they may be present at subtle and subclinical levels. Motor abnormalities, in particular, may be one of the earliest markers observable within the first year [23]. Recent reviews (i.e., [24]) have found evidence for putative ASD biomarkers including gastrointestinal factors [25], immune dysregulation [26, 27], heavy metal toxicity [28–30], neurotransmitter abnormalities [31–33], oxidative stress [34, 35], and elevated levels of p-cresol in small children with ASD [36]. This research suggests that ASD might best be considered to be a multisystem disorder.

Prenatal life and the first three postnatal years are considered to be the optimal time in which to detect and examine the earliest fundamental biological underpinnings of autism [37]. This review specifically focuses on studies published since the beginning of 2009 which investigated whether there were neurological or pathophysiological abnormalities in the first few years of life in individuals later diagnosed with ASD. To our knowledge, this is the first review to focus only on abnormalities within the first few years of life but there have been recent reviews investigating neuroanatomical differences in older children, adolescents, and adults [38, 39]. We will address structural abnormalities (e.g., atypical volume of neural sites, morphology), functional abnormalities (e.g., atypical activation of neural sites), and abnormalities of head circumference. Genetic or environmental aetiologies which may underlie pathophysiological abnormalities are outside the scope of this review [40, 41].

Our understanding of the neural mechanisms that underlie the core symptoms of ASDs has advanced significantly as a result of neuroimaging techniques [42, 43]. Magnetic resonance imaging (MRI) affords the noninvasive in vivo exploration of brain morphology [44] without any adverse effects such as radiation exposure, a crucial feature, particularly when applied to young children [45]. Research on older children through to adulthood with ASD has indicated numerous differences in the neural structures compared to typical developing children and adults. Particularly in the left hemisphere regions, a substantial thinning of the cortex has been observed in individuals with ASD [46] consistent with earlier studies [47]. Increased grey matter in the primary and associative auditory and visual cortex [48] and reductions in regions within the corpus callosum [49] are just some of the findings of brain morphological differences in older individuals with ASD. Subtle differences in both behaviour and brain structure have been discovered within the first 12 months in infants who are later diagnosed with ASD. What is not known is whether any of these subtle differences can be used as an early biomarker to identify infants at-risk of a later ASD diagnosis [50]. Applying behavioural, electrophysiological, and functional neuroimaging methods during the first few years of life in individuals at risk of ASD is essential [51]. The functional brain characteristics of ASD during the time when the behavioural symptoms first arise, around 8–36 months, are largely unknown. Functional magnetic resonance imaging (fMRI) studies have primarily been limited to studies using normal IQ adolescents and adults with ASD [52].

Despite being very much in its infancy, detailed examination of the postmortem brain from individuals with ASD is an area of research which has substantially advanced our understanding of the neurobiological underpinnings of this disorder [53]. Most brain tissues examined have been from adults with ASD, and so our knowledge of the characteristics of the brain in young subjects with ASD is minimal [54].

## 2. Method

Internet-based bibliographic databases (PsycINFO and Web of Knowledge) were searched to access studies which examined neurological differences in individuals with, or later diagnosed with, ASD under the age of three years. Searches were limited to references published from 2009 to the 21st of November 2012 yielding 470 references. Search terms used were “autis*,” “infan*,” “brain,” and “neuro*.” Different ordering of the search criteria entered into either database did not result in any variations in the number of returned abstracts. Duplicates were excluded prior to the retrieval of references. Abstracts for each reference were obtained and screened using the following criteria.

Inclusion criteria:

1. human study population
2. study must involve infants or toddlers under the age of four.

Exclusion criteria:

1. papers not published from January 2009 until 21st November 2012
2. paper not published in English
3. dissertations
4. book reviews.

**Figure 1:**
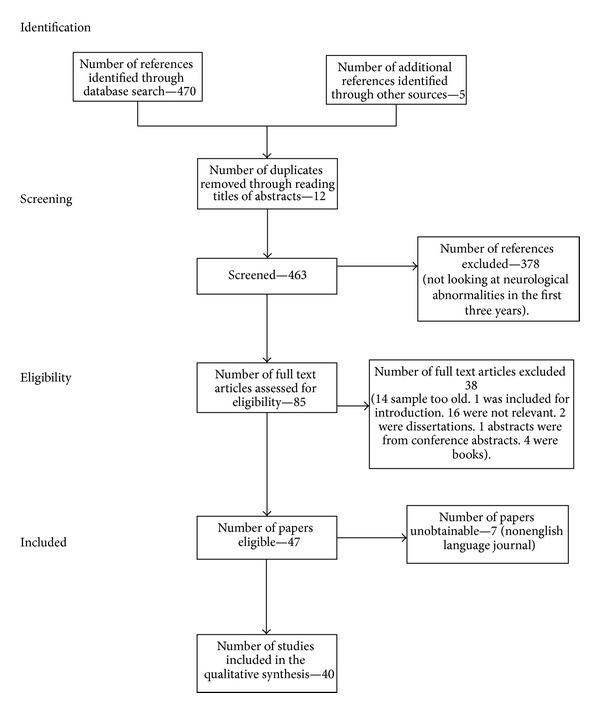
Flowchart showing the process for identifying the relevant studies for this systematic review.

The process of eliminating nonrelevant papers can be seen in the flowchart (following PRISMA guidelines) later (see Figure 1 for flowchart) [55]. We have reviewed studies which contain a mixture of different diseases, albeit all presenting with a somewhat similar autism phenotype.

## 3. Results

Table 1 includes all the studies which investigated neurological differences in individuals with ASD and provides summary detail regarding study characteristics and findings.

### 3.1. Amygdala Abnormalities in Individuals with ASD

Intense interest in the amygdala as the structure predominantly underpinning ASD is not new. The function of the amygdala is related to core clinical features of ASD such as emotion and social behaviour. In addition to the abnormal developmental trajectory of the amygdala, there is a concomitant early overgrowth in ASD. Numerous studies demonstrate amygdala abnormalities in individuals with ASD with increased volumes [94–96] or decreased volumes [97–99] found. Other studies have found no difference [100–102]. The association between abnormalities of amygdala volume and attention to eyes has been found in a study using a sample of older males [99]. Postmortem studies have found quantifiable abnormalities in the amygdala of individuals with ASD [103, 104].

Recent research has emphasised that abnormal developmental trajectory has been relatively under researched in the early years and the age at which abnormal amygdala enlargement begins remains unclear. Schumann et al. [56] measured amygdala volumes on magnetic resonance imaging scans from 89 toddlers at one to five years of age (mean, three years). Toddlers, later diagnosed with ASD (32 boys and nine girls), had a larger right and left amygdala compared with typically developing toddlers (28 boys and 11 girls). In boys, there was a significant positive relationship of amygdala size with severity of clinical impairment. Enlargement in right amygdala volume in males and females and left amygdala volume in females is disproportionate to total cerebral volume at three years. Unlike ASD males, the enlargement in ASD females was associated with severity of social and communication impairments.

Nordahl et al. [57] studied amygdala volumes and total cerebral volumes at two time points in 132 boys (85 with ASD and 47 control subjects with typical development (TD); mean age, 37 months). A year later, longitudinal magnetic resonance images were conducted on 70 participants (45 with ASD and 25 TD controls) and one year growth rates were calculated. Despite no difference in total cerebral volume growth (although the total cerebral volume was significantly enlarged at both time points in the ASD group), at both time points, growth rate and amygdala volume were greater in children with ASD, with enlargement found to be greater at time two. Difference in amygdala volume between the two groups was about 6%, increasing to approximately 9% at time two. Mosconi et al. [58] investigated associations between specific autism behaviours (joint attention) and amygdala volume. Fifty ASD and 33 control (11 developmentally delayed, 22 typically developing) children between 18 and 35 months (two years) of age followed up at 42 to 59 months (four years) of age. Bilateral enlargement of amygdala volume was found in children with ASD. Left amygdala was enlarged proportionately to increases in total tissue volume. A 5% increase in total tissue volume was found in the ASD group and amygdala volumes were enlarged by 16% compared to the control group at the ages of two and four. Between the groups, no differences in the growth trajectories between two and four years of age were found. Interestingly, amygdala enlargement was associated with increased joint attention at the age of four, and while only the right amygdala volume was increased relative to total tissue volume enlargement, the strength of the relationship did not differ when the right and left hemispheres were analysed separately. Lastly, one study emphasised the importance of taking into consideration heterogeneity in studies investigating ASD [59]. Children (between 18 and 42 months) with Fragile x syndrome (FXS) and autism disorder had substantially enlarged caudate volume and smaller amygdala volume. Children with ASD without FXS (i.e., idiopathic autism) had only modest enlargement in their caudate nucleus volumes while enlargement of their amygdala volumes was more pronounced.

### 3.2. Cerebellum Abnormalities in Individuals with ASD

Previous studies have observed reduction in cerebellar grey matter volume in girls with ASD aged between two and six years [105] and increased cerebellar white matter volume (increased by 39%), no enlargement of cerebellar gray matter, and reduced vermis lobules VI-VII in two- and three-year-old ASD children [106]. Sparks et al. [95] found an increase of 7% in the volume of the whole cerebellum in three- and four-year-old ASD children.

Recent research has also found evidence of cerebellum abnormalities. Webb et al. [60] investigated specific cerebellar vermal structures and their association with severity of symptoms and cognitive functioning in children with ASD aged three to four years and found reduced total vermis volumes (vermis lobe VI-VII area) in the ASD children. Neither severity of ASD symptoms nor verbal, nonverbal, or full scale IQ was found to be in correlation with cerebellar measurement. To our knowledge, no studies have investigated cerebellum volumetric differences in children under the age of three within the last three years.

### 3.3. Frontal Cortex Abnormalities in Individuals with ASD

Brain overgrowth is often found in ASD and such overgrowth is commonly found in the prefrontal cortex (PFC) [107–110]. Carper et al. [107] found an anterior-to-posterior gradient of overgrowth, with frontal lobes showing the greatest overgrowth in two to four year olds with ASD. Despite PFC abnormality being considered to underlie some ASD symptoms, the cellular defects that produce the abnormal overgrowth have yet to be discovered.

Studies within the last three years are consistent with earlier findings demonstrating abnormalities within the frontal cortex in individuals with or later diagnosed with ASD. Courchesne et al. [61] examined postmortem prefrontal tissue from seven children with autism and six control male children aged 2 to 16 years and found that children with ASD had 67% more neurons in the PFC compared to controls, including 79% more in dorsalateral-PFC and 29% more in medial-PFC. Brain weight in the ASD cases differed from normative mean weight for age by a mean of 17.6%, while brains in controls differed by a mean of 0.2%.

Both attention and inhibition, previously shown to be associated with frontal cortex activation, were explored in nine to ten month old siblings of children who have been diagnosed with ASD and low-risk control infants [62]. Participants took part in the Freeze-Frame task where infants are encouraged to inhibit looks to peripherally presented distractors whilst looking at a central animation. A subset of sibs-ASD infants had difficulty disengaging attention from a centrally presented stimulus in order to orient to a peripheral stimulus. Lastly, Santos et al. (2011) [63] examined von Economo neurons (VENs) in the frontoinsular cortex (FI), a region which has been put forward as the area which is involved with the integration of internal sensations of bodily arousal, emotional regulation, and goal-directed behaviours. Using a stereological method, Santos et al. (2011) [63] quantified VENs and pyramidal neurons in layer V of FI in postmortem brains of four young patients (aged between 4 and 14 years) with ASD and three age-matched controls and found a significantly higher ratio of VENs to pyramidal neurons in the patients with ASD.

### 3.4. Temporal Cortex Abnormalities in Individuals with ASD

Earlier studies have suggested that failure to develop normal language comprehension is one of the most common early warning signs that a toddler might be at risk for ASD [111, 112] but the neural mechanisms underlying this signature deficit or failure to develop language have yet to be identified. Earlier studies have investigated this using fMRI performed during natural sleep to investigate the brain regions which underlie speech perception [113]. Decreased functional activity in temporal cortices in a small sample (*n* = 12) of two to three year olds with ASD compared with chronological (*n* = 12) and mental age-matched groups (*n* = 11) was found.

A recent study also found lateralised abnormalities of temporal cortex processing of language in ASD. Brain activity in toddlers with ASD (*n* = 40) and typically developing toddlers (*n* = 40), aged between 12 and 48 months, was measured during the presentation of a bedtime story during natural sleep [64]. Deficient left hemisphere response to speech sounds and exhibited abnormally right-lateralised temporal cortex response to language was found in at-risk toddlers who later received a diagnosis of ASD. This was more pronounced in the individuals with ASD when they were three and four years of age. Failed development of language comprehension, known to be one of the earliest markers in ASD, may therefore be the result of very early defects in the superior temporal gyrus which may persist throughout the individual's lifetime.

### 3.5. Differences across a Wide Range of Brain Regions

Abnormal growth was most pronounced in temporal grey matter volumes consistent with earlier findings in children with ASD under the age of two [114] and over [95, 106]. Therefore, abnormal early development of grey matter is linked with ASD (i.e., [115]) in children between two and four years old. Numerous conditions of atypical development can lead to autism, in particular fragile X syndrome (FXS), which is considered to be the most commonly known single-gene cause of autism. Many individuals with FXS also exhibit behaviours common to individuals with ASD.

In a recent study, whole-brain morphometric patterns were examined in young males diagnosed with FXS (*n* = 52; mean age, 2.90 years) or idiopathic autism (iAUT) (*n* = 63, mean age, 2.77 years) as well as typically developing (*n* = 31; mean age, 2.55 years) and idiopathic developmentally delayed controls (*n* = 19; mean age, 2.96 years) [66]. Overall, greater volume was evident in iAUT compared with controls, who, in turn, had greater volume than FXS. Therefore, FXS and iAUT may be associated with distinct neuroanatomical patterns, emphasising the neurobiological heterogeneity of iAUT.

Brain enlargement has been observed in children with ASD as young as two years of age. Hazlett et al. [65] looked at early growth trajectories in brain volume (cerebral grey and white matter) and cortical thickness. At about two years of age, 59 children with ASD and 38 control children were examined using magnetic resonance imaging (MRI). MRI was carried out again approximately 24 months later (when aged 4-5 years; 38 children with ASD; 21 controls). Generalised cerebral cortical enlargement in individuals with ASD at both two and four to five years of age was found (being 9% larger in ASD group). There was no difference in the rate of increase of cerebral cortical growth during this interval between the groups, suggesting that brain enlargement in ASD results from an increased rate of brain growth prior to the age of two years. No cerebellar differences were observed in children with ASD. Despite no difference in cortical thickness, children with ASD had enlargement in both grey and white matter volumes for all cortical lobes (temporal, frontal, and parieto-occipital lobes). However, disproportionate enlargement in temporal lobe white matter was only found in the ASD group after controlling for total brain volume.

Schumann et al. [67] found both cerebral grey and white matter growth abnormalities in individuals with ASD at two and a half years of age. Within cortex, the most significant differences in volume and age-related change took place in anterior regions of the brain (frontal, temporal, and cingulate cortices). Posterior cerebral regions, on the other hand, were less affected with respect to volume and growth trajectory. Abnormal growth was most pronounced in temporal grey matter volumes. Schumann et al. [67] also observed significant gender differences in the longitudinal growth trajectories in numerous brain regions. Compared to controls, in males with ASD, frontal, and temporal lobe grey matter volumes were significantly enlarged and cingulate grey matter grew at a nonlinear rate. Compared to controls, in females with ASD, abnormal brain growth was more diffused and severe with abnormal growth trajectories observed in the total cerebrum, cerebral white, cerebral grey, frontal, and temporal regions. In females with ASD only, there was an enlargement of the cingulate grey supporting this research group's previous hypothesis that males and females with ASD exhibit different “neuroanatomical profiles,” with pathology being more pronounced in females [56]. It has previously been suggested that, compared to females who are later diagnosed with ASD, overgrowth may start earlier in males with ASD.

Wolff et al. [88] prospectively examined white matter fiber tract organisation from six to 24 months in high-risk infants. At 24 months, 28 of the 92 infants met criteria for ASDs. Microstructural properties of white matter fiber tracts (considered to be related with ASDs) were characterised by fractional anisotropy and radial and axial diffusivity. The fractional anisotropy trajectories for 12 of 15 fiber tracts were significantly different between the infants who developed ASDs compared to those who did not. In the infants with ASDs, development for the majority of fiber tracts was characterised by higher fractional anisotropy values at six months followed by slower change over time compared to infants without ASDs [88].

One study investigated structural brain volumes using magnetic resonance imaging across two time points (at two to three and again at four to five years of age). Total brain volumes and regional (lobar) tissue volumes were also examined. The study included 53 boys 18 to 42 months of age with fragile X syndrome (FXS), 68 boys with idiopathic autism (ASD), and a comparison group of 50 typically developing and developmentally delayed controls. Children with FXS had larger global brain volumes compared with controls but were not different than children with idiopathic autism, and the rate of brain growth from two to five years of age was similar to that observed in controls. Children with idiopathic autism were found to have a generalised cortical lobe enlargement, while children with FXS showed specific enlargement in the temporal lobe white matter, cerebellar grey matter, and caudate nucleus but a significantly smaller amygdala [89].

Recognising the neglect of research investigating the neuroanatomical phenotype of female children with ASD (ASDf), Calderoni, Retico, Biagi, Tancredi, Muratori, and Tosetti [90] investigated the anatomic brain structures of a sample entirely composed of ASDf (*n* = 38; two to seven years of age; mean = 53 months; SD = 18) compared to 38 female age and nonverbal IQ matched controls. Whole brain volumes of each group were compared using voxel-based morphometry (VBM) with diffeomorphic anatomical registration through exponentiated lie algebra (DARTEL) procedure, allowing the authors to create a study-specific template. First, the between-group whole-brain and brain-segment volume comparison revealed a total intracranial volume (TIV) enlargement of approximately 5% in female children with ASD with respect to age and NVIQ matched controls. Second, the conventional VBM analysis showed evidence of an increased GM volume in a specific region of the left superior frontal gyrus of ASDf. Third, the implementation of the SVM analysis on the GM segments obtained in the VBM-DARTEL preprocessing highlighted a more complex circuitry of increased cortical volume in ASDf, involving bilaterally the SFG and the right temporoparietal junction (TPJ), compared to controls [90].

Lastly, some studies have found no evidence of abnormalities across brain structure in individuals with ASD in the early years. For instance, thirty-four children with ASD and 13 developmentally delayed children without ASD, between two and seven years of age (matched on age and developmental level), participated in an MRI study to investigate volumes of cranium, total brain, cerebellum, grey and white matter, ventricles, hippocampus, and amygdale [91]. No significant differences in volumes of intracranium, total brain, ventricles, cerebellum, grey or white matter or amygdala and hippocampus between the ASD group and the developmentally delayed group were found. The important suggestion arising from these findings is that higher intellectual functioning was not found to be associated with a relatively larger brain volume in children with ASD, therefore relative brain enlargement may not be beneficial to individuals with ASD [91]. This merits further research in this area. Also, an MRI study examining head circumference, brain volume and radiologic abnormalities in a group of six-month-old infants at high risk for autism (*n* = 98) compared to infants without family members with autism (*n* = 36) found no significant group differences [92].

### 3.6. Relationship between Increased Head Circumference (HC) and Somatic Growth in ASD

Accelerated brain growth is a well known and intriguing biological feature in children with ASD [72, 116, 117]. A recent study suggested that, in fact, at the total population level, macrocephaly is uncommon in ASD [118]. Evidence of accelerated head circumference (HC) or macrocephaly and body growth during infancy in children with ASDs is well supported in the literature, although variation in the timing of acceleration across studies exists [106, 109, 119]. Such accelerated growth has even been suggested as an early biological indicator of ASD within the first 12 months of life [120, 121]. Research investigating whether abnormally large HC during the early years can be a reliable indicator of ASD is supported by findings that HC during the early years more accurately reflects brain volume than that during adolescence and a crucial factor for the analysis of ASD onset is the timing of the increase in HC in infancy and toddlerhood [120, 122, 123]. Emergence of brain organisation and connectivity differences in high risk infants occur at the same time as observations of accelerated head growth have been found in children with ASD [124]. Accelerated head growth in ASD has been argued to be the result of an increase in general body growth [123, 125, 126]. HC trajectories were still accelerated in children with ASD even after correcting for body length and height measurements [120, 123]. Despite a large number of studies investigating head circumference in young children with ASD, research carried out over the last three years have produced mixed results.

More recent studies have investigated HC within the first few years of life. Rommelse et al. [68] measured HC, height and weight throughout the first 19 months of life in 129 children with ASD and 59 children with non-ASD psychiatric disorders. Fifty-nine children (46 male and 13 female) with non-ASD psychiatric disorders (Psychiatric controls, PC) also participated: 39 had a psychiatric disorder other than ASD (such as ADHD, oppositional defiant disorder, communication disorder), 12 had a diagnosis according to the DC: 0-3R (2005; such as regulation problems), and eight had mental retardation without any psychiatric comorbidity. Similar abnormal patterns of growth compared to population norms were found in both groups, especially regarding height and HC in relation to height. Abnormal HC growth may actually be common to psychiatric disorders, rather than ASD specifically, questioning the use of HC growth as a marker for ASD. However, the most apparent difference was that the children with ASD only showed an increased HC relative to height up to two months of age, an increase not found in the PC group at this age.

Muratori et al. [69] used anthropometric measurements (HC, body height, and body weight) obtained at birth (T0), 1-2 months (T1), 3–5 months (T2) and 6–12 months (T3) to investigate HC development during the first year. At T2 and T3, HC was significantly larger in the ASD group (*n* = 50) compared to the typically developing group (*n* = 100). Weight was significantly less in ASD subjects from 1-2 months onwards. After controlling for weight and height, an excessive rate of HC growth from birth was found in the individuals with ASD consistent with an earlier study by Fukumoto et al. [70] which compared 280 children with ASD. Increases in HC growth from 3 to 12 months, in height from 3 to 9 months and in body weight from three-six months and 12 months were found in the males with ASD. Increases in HC, body height, and body weight were only observed at three months in the females with ASD. Only the HC in the male ASD group were significantly increased from six to nine months after birth, reaching a peak at six months after birth after correcting for height, age and weight. Chawarska et al. [71] examined whether HC growth in ASD is independent of height and weight growth during infancy and also whether there is any association between HC growth from birth to 24 months and measures of cognitive functioning (social, verbal, cognitive and adaptive functioning) taken at two years of age. Boys diagnosed as having autism disorder (*n* = 64), pervasive developmental disorder (not otherwise specified) (*n* = 34), global developmental delay (*n* = 13), and other developmental problems (*n* = 18) and typically developing boys (*n* = 55) were compared. Boys with ASD were significantly longer by 4.8 months, had greater HC by age 9.5 months and weighed more by age 11.4 months, compared to the typically developing boys. No other clinical groups displayed overgrowth and boys with ASD who were in the top 10% of overall physical size in infancy exhibited more severe social deficits and lower adaptive functioning at two years.

Rapid head growth has been suggested as a potential risk factor for regressive autism [72]. Using a large sample of two to four year old boys and girls with ASD (*n* = 53, no regression (nREG); *n* = 61, regression (REG)) and a control group of age-matched typically developing controls (*n* = 66). Retrospective measurements of HC from birth through to 18 months of age were reviewed and abnormal brain enlargement was most commonly found in boys with regressive autism whereas brain size in boys without regression were similar to controls. HC in boys with regressive autism was normal at birth but deviated from normal growth trajectories (other groups) around the age of four to six months. No brain size differences in girls with autism (*n* = 22, ASD; *n* = 24, controls) were found. Nordahl et al. [72] argue that distinct neural phenotypes are linked with different onsets of ASD. For boys with regressive autism, divergence in brain size occurs well before loss of skills are typically observed. Investigating age-specific anatomical abnormalities in individuals with ASD, Courchesne et al. [37] measured age-related changes in brain size in ASD and control participants (between 12 months and 50 years of age) based on the analyses of 586 longitudinal and cross-sectional MRI scans. Findings revealed evidence of overgrowth throughout infancy and toddlerhood in both boys and girls with ASD which was subsequently followed by an accelerated rate of decline in size.

While the studies discussed so far indicate abnormalities in HC in individuals with ASD, numerous studies have found no evidence of such differences. In a nationally representative, community-based sample of children with and without ASDs, derived from the Early Childhood Longitudinal Study Birth Cohort, Barnard-Brak et al. [73] followed about 9,000 children across three time points (9, 24, and 36 months) to see the HC growth trajectory over this time. No difference in HC at any of the three time points in the children with ASDs were found. Whitehouse et al. [74] were the first to examine foetal HC growth prospectively in children with ASD (*n* = 14) who were each matched with four control participants (*n* = 56) on a variety of factors which can have an effect on foetal growth. HC was measured using ultrasonography at about 18 weeks gestation and also at birth using a paper tape-measure. Overall body size was indexed by foetal femur-length and birth length. This study found no difference in HC at either time-point between the groups.

A retrospective study obtained serial head orbitofrontal circumference measurements taken from 48 sibling pairs in which one (*n* = 28) or both (*n* = 20) siblings were affected by an ASD and 85 control male sibling pairs over 15 time points starting from birth to 36 months [75]. There was a significant acceleration of head growth in individuals with ASD compared to controls. The study also showed that infant HG trajectory may be endophenotypic but was not a reliable indicator of risk of ASD among siblings of ASD in this study. Gray et al. [76] measured HC at birth and rate of change in HC in young children with autism (*n* = 86) and children with developmental delay without autism (*n* = 40) and found no differences between the group of children with both ASD and developmental delay compared with the group with developmental delay alone. However, compared with normative data, children with ASD had significantly smaller HCs at birth and significantly larger HC at 18.5 months of age with no difference in the HCs of children with ASD and developmental delay and children with developmental delay only indicating that HC measurement has limited reliability in terms of its use as an early indicator for ASD. Lastly, in the first study to examine head growth in children who later lose their diagnoses of ASD, Mraz et al. [77] measured HC, length, and weight growth during infancy for 24 children who maintained their diagnoses, 15 children who lost their diagnoses, and 37 typically developing controls. Compared to controls, HC and weight growth were significantly larger in both ASD groups (birth to 25 months) and there were no significant differences between ASD groups.

### 3.7. Electrophysiological Functioning Differences in Individuals with ASD

By around one year, infants at high risk for ASD display behavioural deficits in social development at increased rates compared to low-risk infants [127, 128]. These subtle brain function signatures (atypical neural electrophysiological responses) in the first few years of life may provide an early indicator to later development of complex neurodevelopmental disorders such as ASD. Earlier research, which primarily has been limited to older children, suggests that early detection of abnormalities in electroencephalography (EEG) signals may be used as an early biomarker for developmental cognitive disorders [129]. Atypicalities in face and object processing in children and adults with ASDs have previously been shown in three to four year olds with ASD [130] and adults with ASD [131]. Since indicators of brain function may serve as potentially sensitive predictors of ASD and atypical eye contact are characteristic of this syndrome [132], studies have previously investigated whether neural sensitivity to eye gaze during infancy is associated with later autism outcomes [133, 134] and atypical eye gaze processing in children and adults with ASD have been shown.

A more recent study investigated whether such atypicalities reflect an early genetically mediated risk factor [78] by measuring cortical responses to face/object processing in ten month old high-risk infants (siblings of an older sibling diagnosed with ASD) using event-related potentials (ERPs). Latencies and amplitudes of four ERP components (P100, N290, P400, and Nc) were compared between 20 high-risk infants and 20 low-risk control subjects. The low-risk group displayed faster responses to faces compared to object stimuli (P400) which was not observed in the high-risk group. Conversely, faster responses to objects rather than faces in high-risk but not low-risk infants (N290) were shown. Responses to objects were also faster in high-risk compared to low-risk infants (both N290 and P400). Overall there were significantly less hemispheric asymmetries exhibited in the high-risk compared to the low-risk group.

Luyster et al. [79] investigated whether high-risk infants might also exhibit atypical neural responses to social stimuli. The face-sensitive N290/P400 complex and the Nc, associated with the allocation of attention, were studied. Thirty-two 12-month-old infants at high risk of ASD and 24 low-risk control infants were presented with familiar and unfamiliar faces. There were no significant group differences in the neural response to faces. A more negative Nc to unfamiliar faces than to familiar ones across both groups were displayed, thus indicating that infants recruited more attentional resources when presented with an unfamiliar face compared with a familiar one. This lack of differentiation between familiar and unfamiliar stimuli in high-risk infants is consistent with the findings reported earlier by McCleery et al. [78].

In light of previous findings demonstrating atypical eye gaze processing in children and adults with ASD, Elsabbagh et al. [80] recently examined the neural correlates of direct and averted gaze in infant siblings of children diagnosed with ASD (Sib-ASD). Nineteen siblings of children diagnosed with ASD (sib-ASD) were compared with 17 control infants with no family history of ASD (mean, ten months) on their response to direct versus averted gaze in static stimuli. Prolonged latency of the occipital P400 event-related potentials component in response to direct gaze was exhibited in the sib-ASD group compared to control infant. However, there was no difference between the groups in the P400 latency for averted gaze. The N290 is also a component sensitive to attentional modulation in infants [135]. While the control group showed no difference in latency values between Direct and Averted gaze, the sib-ASD group had a tendency to respond faster to the Averted relative to the Direct gaze condition. Neural sensitivity to eye gaze in infancy may therefore serve as an early predictor of ASD later in toddlerhood. Elsabbagh et al. [8] examined whether neural sensitivity to eye gaze during infancy is associated with later diagnosis of ASD and outcomes. Infants at high familial risk for ASD (*n* = 54) and a comparison group of infants at low risk (*n* = 50) took part in a study which recorded electrophysiological brain responses (ERPs) while six to ten month old infants viewed faces with dynamic eye gaze directed either towards them or away from them. Characteristics of ERP components evoked in response to dynamic eye gaze shifts during infancy were associated with ASD diagnosis at 36 months. Despite the rarity of observing behavioural symptoms or signs of ASD in the first year, atypical brain function during this first year distinguished the group of infants who were later diagnosed with ASD [8].

In another study the usefulness of two methods, regularised discriminant function analyses and support vector machines, were shown by reanalysing an ERP dataset of infants from a study discussed earlier in this section [80]. Stahl, Pickles, Elsabbagh, Johnson, and The BASIS Team [87] found supportive evidence that these classification methods can increase the discriminative power of ERP measurements. Using cross-validation, both methods successfully discriminated at above chance levels between groups of infants at high and low risk of a later diagnosis of autism. However, infants could only be discriminated in the direct gaze condition, not in the averted gaze condition [87].

One study investigated whether infant siblings of children with ASD (sibs-ASD) process familiar and novel faces differently from typical infants [81]. ERPs were recorded in 35 infants, approximately nine months 15 days old (20 typical infants, 15 sibs-ASD) using an oddball paradigm presenting photographs of infants' mothers and an unfamiliar female. No differences were revealed in the distribution, number, or duration of fixations between the groups. Both groups differentiated between mothers and strangers. However, there was a delayed ERP response to the stranger face (as evidenced by the latency of the P400 response) in the typical infants only. Another eye tracking study in two to four year old toddlers with ASD found atypical face scanning to become more pronounced with age [83]. Toddlers with ASD looked increasingly away from faces with age (from testing at two years and again at four years) and atypically attended to key features within the face and demonstrated impaired ability to recognise faces at both ages.

Key and Stone [82] examined whether, on average, nine month old infants, compared to infants at high risk for ASD, process facial features (eyes, mouth) differently and whether such differences were related to the infants' social and communicative skills. Eye tracking and visual event-related potentials (ERPs) were recorded in 35 infants (20 average-risk typical infants, 15 high-risk siblings of children with ASD) while they viewed photographs of a smiling unfamiliar female face. On 30% of the trials, the eyes or the mouth of that face was replaced with corresponding features from a different female. No group differences in the number, duration, or distribution of fixations were evident and all infants looked at the eyes and mouth regions equally. Findings from ERP analysis showed that all infants detected eye and mouth changes but did so using different brain mechanisms. Facial feature changes were related to changes in activity of the face perception mechanisms (N290) for the average-risk group only. For all infants, correlations between ERP and eye-tracking measures indicated that larger and faster ERPs to feature changes were associated with fewer fixations on the irrelevant regions of stimuli. Size and latency of the ERP responses correlated with parental reports of receptive and expressive communication skills.

Bosl et al. [84] adopted modified multiscale entropy (mMSE) computed on the basis of resting state EEG data, to determine whether typically developing children can be distinguished from a group of infants at high risk for ASD (older sibling with ASD). To the author's knowledge, this is the first study to look into connectivity changes across time in young children at high risk for developing autism. It shows differences in resting brain state entropy, possibly indicating a biomarker for risk for a complex neurodevelopmental disorder. Classification was computed separately within each age group from six to 24 months. Data was collected from a total of 143 sessions and from 79 individuals. Multiscale entropy appears to go through a different developmental trajectory in infants at high risk for ASD than it does in typically developing controls with differences being most marked at ages nine to 12 months. Lastly, Webb et al. [85] investigated neural responses to familiar and unfamiliar faces in twenty-four children with ASD (18 to 47 months old) compared with responses of thirty-two typically developing children (12 to 30 months old). Delayed development in the individuals with ASD was indicated since neural responses to faces in this group of children resembled those observed in younger typically developing children. Interestingly, electrophysiological responses to faces were associated with parental report of adaptive social behaviours for children with ASD and typically developing children.

Lastly, in a large case control study, a stable pattern of EEG spectral coherence was found to distinguish children with ASD from neurotypical controls in the subgroup aged between two and four years [93].

### 3.8. Interhemispheric Synchronisation in Individuals with ASD

Connectivity studies during the very early years in ASD are few in number. Using fMRI data, Dinstein et al. [86] found disrupted synchronisation in the spontaneous cortical activity of 29 naturally sleeping toddlers with ASD (1–3.5 years old) which was not evident in the toddlers with language delay or the typical development group. In toddlers with ASD, significantly weaker interhemispheric synchronisation (weak “functional connectivity” across the two hemispheres) was revealed in the inferior frontal gyrus (IFG) and superior temporal gyrus (STG), two areas commonly associated with language production, and comprehension. There was also a significant inverse relationship between interhemispheric synchronisation strength and autism severity. Strength of interhemispheric synchronisation was positively correlated with verbal ability. Investigation of neural synchronisation may be useful as a diagnostic measure to aid growing efforts of identifying ASD during infancy [9].

## 4. Future Directions

Further research delineating the neurological mechanisms underlying ASD is of clinical importance [43]. Within the last decade in particular, there has been a substantial increase in research focused on understanding the biological mechanisms underlying ASD; however, many fundamental issues remain. For instance, the causes of ASD, the specific brain regions most impacted by ASD, and why are there more males with ASD and what are the underlying mechanisms involved that produce neurological gender differences [54]. Given that behavioural markers of ASD within the first year of life are subtle in nature, it may be that neurological methods may prove to be more sensitive at this early stage in identifying and quantifying risk. Research investigating whether a combination of risk markers early in infancy is more effective than individual markers of risk in predicting diagnostic outcomes for ASD is necessary [7].

Due to the numerous factors which could be contributing to brain volume, further research is needed to explain what is involved in producing the unusual amygdala growth trajectory as well as other areas which have been found to be enlarged in individuals with ASD. What we do know is the importance of taking into account both the age and gender of the individual when interpreting findings in volumetric studies of ASD [56]. Behavioural correlates of different amygdala growth trajectories**  **is another potentially interesting avenue for research. One hypothesis would be that children with ASD who exhibit accelerated amygdala growth might show higher anxiety levels [57].

Further research investigating the association between HC growth rates and ASD is necessary since the majority of research so far has been limited by small sample sizes and by an absence of necessary group comparisons, such as developmentally delayed children. Characteristics of the subgroup of children who exhibit accelerated head growth within the first 18 months of life needs to be investigated using a longitudinal approach [42]. Future studies are required to examine whether impaired interhemispheric synchronisation in putative language areas plays a causal role in generating autism behavioural symptoms [86].

Despite the advances in our knowledge of neurological abnormalities in the brain of individuals with ASD, numerous challenges**  **still remain, for instance, the heterogeneity of symptoms, symptom severity, differences in IQ, total brain volume, and psychiatric comorbidity [42]. Lastly, research into the plasticity in autism has yet to be carried out but it would be invaluable to our understanding of the possibility of altering the course of brain development in individuals with ASD [136].

## 5. Conclusion

With growing interest in identifying earlier methods for detecting ASD, these studies are paving the way towards the development of noninvasive, brain-based screening methods that could potentially detect differences prior to behavioural emergence [137] which would constitute an important scientific breakthrough [138]. Cross-disciplinary advances have contributed to a “more optimistic outcome” for individuals with ASD [139] and the development of new methods for early detection and more effective treatments [11]. Since we are still not aware of the protective factors, ethical issues concerning the implementation and clinical recommendations based on biomarker measures need to be carefully considered [140]. The importance of cross disciplinary research, in particular combining findings from the behavioural and neurological fields, is emphasised by Happé et al. [141] when they stated that “abandoning the search for a single cause for a single entity of autism may also mean abandoning the search for a single “cure” or intervention.”

## 6. Conflict of Interests 

The authors declare that there is no conflict of interests regarding the publication of this paper.

## Figures and Tables

**Table 1:** Studies investigating neurological differences in individuals with ASD: study characteristics and findings.

Author	Age of sample	Sample size	Aim of the study	Findings
Schumann et al. [56]	1–5 years (mean age 3 years). Each child returned at approx. 5 years for final clinical evaluation.	41 autism, 9 PDD-NOS, and 39 TD controls.	Amygdala volume	Toddlers with ASD diagnosis (32 boys, 9 girls) had a larger right and left amygdala compared with typically developing toddlers (28 boys, 11 girls).

Nordahl et al. [57]	Time 1—mean age 37 months. Time 2—one years later.	At time 1—132 boys (85 with ASD and 47 controls with TD). At time 2—70 boys (45 with ASD and 25 TD controls).	Amygdala volumes and total cerebral volumes (TCV).	Despite no difference in TCV growth (although the TCV was significantly enlarged at both time points in the ASD group), at both time points, growth rate and amygdala volume were greater in children with ASD, with enlargement found to be greater at time two.

Mosconi et al. [58]	18–35 months (2 years). Followed-up at 42–59 months (4 years).	50 ASD and 33 controls (11 DD and 22 TD).	Investigated associations between specific ASD behaviours (joint attention) and amygdala volume.	Bilateral enlargement of amygdala volume was found in children with ASD. There was a disproportionate right amygdala volume enlargement compared to total tissue volume. Amygdala enlargement was associated with increased JA at age four years.

Hazlett et al. [59]	18–42 months	52 fragile x syndrome (FXS); 63 autism, 19 DD, and 31 TD.	Caudate nucleus volume and amygdala volume.	Children with FXS and autism disorder had substantially enlarged caudate volume and smaller amygdala volume. Children with ASD without FXS (i.e., idiopathic autism) had only modest enlargement in their caudate nucleus volumes while enlargement of their amygdala volumes were more pronounced.

Webb et al. [60]	3-4 years	45 children with ASD, 14 children with DD, and 26 children with TD.	Cerebellar vermal structures and their association with severity of symptoms and cognitive functioning in children with ASD.	Reduced total vermis volumes (vermis lobe VI-VII area) in the ASD children. No correlation was found between cerebellar measurements and severity of ASD symptoms nor verbal, nonverbal, or full scale IQ.

Courchesne et al. [61]	2–16 years	7 ASD and 6 control male children.	Postmortem prefrontal tissue	Children with ASD had 67% more neurons in the PFC compared to controls, including 79% more in dorsalateral-PFC and 29% more in medial-PFC.

Holmboe et al. [62]	9 to 10 month old	31 siblings of children who have been diagnosed with ASD and 33 low-risk control infants.	Frontal cortex functioning using a task exploring attention and inhibition.	A subset of sibs-ASD infants had difficulty disengaging attention from a centrally presented stimulus in order to orient to a peripheral stimulus indicating atypical frontal cortex functioning in the infant broader autism phenotype.

Santos et al. [63]	4–14 years	Postmortem brains of 4 young patients with ASD and 3 aged-matched controls.	von Economo neurons in the frontoinsular cortex using postmortem brain tissue.	A significantly higher ratio of VENs to pyramidal neurons was found in the sample of ASD patients.

Eyler et al. [64]	12–48 months	40 with ASD and 40 TD.	Lateralised abnormalities of temporal cortex processing of language.	Deficient left hemisphere response to speech sounds and exhibited abnormally right-lateralised temporal cortex response to language was found in at-risk toddlers who later received a diagnosis of ASD. Difference becomes greater with age.

Hazlett et al. [65]	About two years of age. MRI was carried out again approximately 24 months later (when aged 4-5 years; 38 children with ASD; 21 controls).	59 children with ASD and 38 control children. Thirty-eight children with ASD and 21 comparison cases were examined at the follow-up visit.	Early growth trajectories in brain volume (cerebral gray and white matter) and cortical thickness.	Generalised cerebral cortical enlargement in individuals with ASD at both two and four to five years (being 9% larger in ASD group). Despite no difference in cortical thickness, children with ASD had enlargement in both grey and white matter volume for all cortical lobes (temporal, frontal and parieto-occipital lobes). Disproportionate enlargement in temporal lobe white matter only was found in the ASD group after controlling for total brain volume.

Hoeft et al. [66]	FXS group—mean age 2.9 years. idiopathic autism (iAUT)—mean age 2.77 years. Typical developing—mean age 2.55 years. Idiopathic developmentally delayed controls—mean age 2.96 years.	52 males diagnosed with FXS. 63 with Idiopathic autism (iAUT). 31 TD. 19 idiopathic DD controls.	Whole-brain morphometric patterns.	Greater volume was evident in iAUT compared with controls, who in turn had greater volume than FXS. Frontal and temporal grey and white matter regions often implicated in social cognition, including the medial prefrontal cortex, orbitofrontal cortex, superior temporal region, temporal pole, amygdala, insula, and dorsal cingulum were abnormal in FXS and iAUT.

Schumann et al. [67]	1.5 years–5 years of age. Mean, 30 months plus or minus 10 months).	41 toddlers who received a confirmed diagnosis of autism disorder at 48 months of age and 44 TD controls.	Cerebral gray and white matter growth.	Cerebral grey and white matter growth abnormalities in individuals with ASD. Within cortex, the most significant differences in volume, and age-related change took place in anterior regions of the brain (frontal grey, temporal grey, and cingulate grey cortices). Posterior cerebral regions less affected. Abnormal growth most pronounced in temporal grey matter volumes.

Rommelse et al. [68]	0–19 months	129 children with ASD and 59 children with non-ASD psychiatric disorders.	Head circumference, height, and weight.	Similar abnormal patterns of growth compared to population norms were found in both groups. Abnormal HC growth may actually be common to psychiatric disorders, rather than ASD specifically. However, the most apparent difference was that the children with ASD showed an increased HC relative to height up to two months of age, an increase not found in the PC group at this age.

Muratori et al. [69]	Birth (TO), 1-2 months (T1), 3–5 months (T2), and 6–12 months (T3).	50 with ASD and 100 TD.	Head circumference, body height, and body weight.	Weight was significantly less in ASD subjects compared to healthy infants from 1-2 months onwards. After controlling for weight and height, excessive rate of HC growth from birth was found in the individuals with ASD.

Fukumoto et al. [70]	3–12 months	280 children with ASD.	Head circumference, body height, and body weight.	Increases in HC growth from 3–12 months, in height from 3–9 months, and in body weight from 3 to 6 and 12 months were found in the males with ASD. Increases in HC, body height and body weight were only observed at three months in the females with ASD. Only HC in the male ASD group was significantly increased from 6–9 months after birth.

Chawarska et al. [71]	0–24 months	Autism disorder (*n* = 64), PDD-NOS (*n* = 34), global developmental delay (*n* = 13), and other developmental problems (*n* = 18), and TD boys (*n* = 55).	Head circumference growth in ASD, height, and weight growth. Investigate association between head circumference growth from birth to 24 months and measures of cognitive functioning.	Boys with ASD were found to be significantly longer by 4.8 months, had greater HC by age 9.5 months and weighed more by age 11.4 months, compared to the typically developing boys. No other clinical groups displayed an overgrowth. Boys with ASD in the top 10% of overall physical size in infancy displayed more severe social deficits and lower adaptive functioning at 2 years.

Nordahl et al. [72]	2–4 years	Boys and girls with ASD (*n* = 53, no regression (nREG); *n* = 61, regression (REG)) and a control group of age-matched typically developing controls (*n* = 66).	Total brain volume (rapid head growth).	Abnormal brain enlargement was most common in boys with regressive autism. Brain size in boys without regression was similar to controls. Head circumference in boys with regressive autism was normal at birth but deviated from normal growth trajectories (other groups) around the age of 4–6 months. No brain size differences in girls with autism (*n* = 22, ASD; *n* = 24, controls).

Courchesne et al. [37]	12 months and 50 years of age for the typical group and 2–50 years for the ASD group.	259 ASD subjects and 327 TD subjects.	Brain size based on the analyses of 586 longitudinal and cross-sectional MRI scans.	Evidence of overgrowth throughout infancy and the toddlerhood in both boys and girls with ASD which was subsequently followed by an accelerated rate of decline in size.

Barnard-Brak et al. [73]	3 time points (9, 24 and 36 months)	About 9,000 children.	Head circumference growth trajectory.	No difference in HC at any of the 3 time points in the children with ASDs.

Whitehouse et al. [74]	18 weeks gestation and also at birth	14 children with ASD were matched with four control participants (*n* = 56).	Head circumference was measured using ultrasonography at about 18 weeks gestation and also at birth using a paper tape-measure. Overall body size was indexed by foetal femur-length and birth length.	No difference in head circumference at either time-point between the groups.

Constantino et al. [75]	15 time points starting from birth to 36 months of age	48 sibling pairs in which one (*n* = 28) or both (*n* = 20) sibs were affected by an ASD and 85 control male sibling pairs	Serial head orbitofrontal circumference measurements.	Significant acceleration of head growth in individuals with ASD compared to controls. The study also showed that infant HG trajectory may be endophenotypic but was not a reliable indicator of risk of ASD among siblings of ASD in this study.

Gray et al. [76]	Birth and 18.5 months.	Children with autism (*n* = 86) and children with DD without autism (*n* = 40).	Head circumference at birth and rate of change in head circumference.	No differences between the group of children with both ASD and developmental delay compared with the group with developmental delay alone. However, when compared with normative data, children with ASD had significantly smaller HCs at birth and significantly larger HC at 18.5 months of age.

Mraz et al. [77]	0–25 months	24 children who maintained their diagnoses, 15 children who lost their diagnoses and 37 TD controls.	Head circumference, length and weight growth.	Compared to controls, HC and weight growth were significantly larger in both ASD groups and there were no significant differences between ASD groups.

McCleery et al. [78]	10 months	20 high-risk infants (siblings of an older sibling diagnosed with ASD) and 20 low-risk control subjects.	Cortical responses to face/object processing using event-related potentials.	The low-risk group displayed faster responses to faces compared to object stimuli (P400) which was not observed in the high-risk group. Conversely, faster responses to objects than faces in high risk but not low-risk infants (N290). Right hemisphere advantage (greater hemispheric asymmetry) in the typical that was not found in the ASD group.

Luyster et al. [79]	12 months	32 infants at high-risk of ASD and 24 low-risk control infants.	Atypical neural responses to social stimuli.	No significant group differences in the neural response to faces. Trend for the low-risk group to exhibit more marked differential response to familiar and unfamiliar faces (in the anticipated direction) compared with high-risk infants.

Elsabbagh et al. [80]	Mean age—10 months	Nineteen infant siblings of children diagnosed with ASD and 17 control infants with no family history of ASD.	Neural correlates of direct and averted gaze.	Prolonged latency of the occipital P400 event-related potentials component in response to direct gaze was exhibited in the sib-ASD group compared to control infant. No difference between the groups in the P400 latency for Averted gaze.

Elsabbagh et al. [8]	6–10 month. About 18 to 30 months later, these children were clinically assessed for ASD.	Infants at high familial risk for ASD (*N* = 54) and infants at low risk (*n* = 50).	Neural sensitivity to eye gaze.	Characteristics of ERP components evoked in response to dynamic eye gaze shifts during infancy were associated with ASD diagnosis at 36 months.

Key and Stone [81]	Approximately nine months 15 days old	20 typical infants and 15 infant siblings of children diagnosed with ASD.	Speed of processing of novel versus familiar faces using event related potentials and eye tracking.	Both infant groups demonstrated the ability to differentiate between mothers and strangers, as shown in the amplitude modulations of posterior N290/P400 and frontal/central Nc responses. However there was a delayed ERP response to the stranger face (as evidenced by the latency of the P400 response) in the typical infants only.

Key and Stone [82]	Mean age of High-Risk group was 9.01 (0.34).	35 infants (20 average-risk typical infants, 15 high-risk siblings of children with ASD).	To investigate whether infants at high risk for ASDs process facial features (eyes, month) differently. Also, whether this is associated with social and communicative skills.	All infants detected eye and mouth changes. However, different brain mechanisms were used. Facial feature changes were related to changes in activity of the face perception mechanisms (N290) for the average-risk group only.

Chawarska and Shic [83]	Testing at 2 years and then again at 4 years. Chronological age (months) for ASD at time 1 26.9 (6.2) and time 2 46.4 (6.4). For control group mean age at time 1 26.3 (6.5) and time 2 46.3 (4.3).	44 children with ASD and 30 TD controls.	Eye tracking—atypical face scanning.	Toddlers with ASD looked increasingly away from faces with age and atypically attended to key features within the face. They also demonstrated at both ages impairment in the ability to recognise faces.

Bosl et al. [84]	6–24 months	46 high risk for ASD, defined on the basis of having an older sibling with a confirmed diagnosis of ASD and 33 controls.	Modified multiscale entropy (mMSE) computed on the basis of resting state EEG data.	Multiscale entropy appears to go through a different developmental trajectory in infants at high risk for ASD than it does in typically developing controls with differences being most marked at ages 9–12 months.

Webb et al. [85]	ASD group—18–47 months. Typically dev.—12–30 months.	24 children with ASD and 32 TD children.	Neural responses to familiar and unfamiliar faces.	Delayed development in the individuals with ASD was indicated since neural responses to faces in this group of children resembled those observed in younger typically developing children.

Dinstein et al. [86]	1–3.5 years	72 toddlers in total. Broken down in study: All toddlers (12–46 months)—Autism (*n* = 29), Control (*n* = 30) and language delay (*n* = 13). Young toddlers (12–24 months)—Autism (*n* = 12), Control (*n* = 16) and Language Delay (*n* = 11).	Spontaneous cortical activity of naturally sleeping toddlers with autism. fMRI data.	Toddlers with autism exhibited significantly weaker interhemispheric synchronization (i.e., weak ‘‘functional connectivity” across the two hemispheres) in putative language areas.

Stahl et al. [87]	10 month olds	10 infant at risk of ASD.	Aim was to discuss the use of machine learning and discrimination methods and their possible application to the analysis of infant event-related potential (ERP) data.	Classification methods (regularised discriminant function analyses and support vector machines) can increase the discriminative power of ERP measurements. Using cross-validation, both methods successfully discriminated at above chance levels between groups of infants at high and low risk of a later diagnosis of autism. However, infants could only be discriminated in the direct gaze condition, not in the averted gaze condition.

Wolff et al. [88]	6 to 24 months in high-risk infants.	92 high-risk infant siblings from an ongoing imaging study of ASD.	The authors prospectively examined white matter fiber tract organisation from 6 to 24 months in high-risk infants who developed ASD by 24 months.	The fractional anisotropy trajectories for 12 of 15 fiber tracts were significantly different between the infants who developed ASDs compared to those who did not.

Hazlett et al. [89]	68 boys with idiopathic autism (ASD). 18 to 42 months of age.	53 boys with fragile X syndrome (FXS), 68 boys with idiopathic autism (ASD), and a comparison group of 50 typically developing and developmentally delayed controls.	Total brain volumes and regional (lobar) tissue volumes were also examined.	Children with idiopathic autism were found to have a generalised cortical lobe enlargement.

Calderoni et al. [90]	Female children with ASD (ASDf)—2–7 years.	ASDf (*n* = 38) compared to 38 female age and non verbal IQ matched controls.	Aim to investigate the neuroanatomical phenotype of female children with ASD.	The between-group whole-brain and brain-segment volume comparison revealed a total intracranial volume (TIV) enlargement of approximately 5% in female children with ASD. The conventional VBM analysis showed evidence of an increased GM volume in a specific region of the left superior frontal gyrus of ASDf. The implementation of the SVM analysis on the GM segments obtained in the VBM-DARTEL pre-processing highlighted a more complex circuitry of increased cortical volume in ASDf, involving bilaterally the SFG and the right temporo-parietal junction (TPJ).

Zeegers et al. [91]	Between 2–7 years.	34 children with ASD and 13 developmentally delayed children without ASD, (matched on age and developmental level).	To investigate volumes of cranium, total brain, cerebellum, grey and white matter, ventricles, hippocampus, and amygdale.	No significant differences in volumes of intracranium, total brain, ventricles, cerebellum, grey or white matter or amygdala and hippocampus between the ASD group and the developmentally delayed group were found.

Hazlett et al. [92]	6 month-old infants at high risk for ASD	Infants at high risk for ASD (*n* = 98) compared to infants without family members with ASD (*n* = 36).	MRI study examining head circumference, brain volume and radiologic abnormalities.	No group differences.

Duffy and Als [93]	Between 2–4 years	The 2- to 12-year-old subsample consisted of 430 ASD- and 554 C-group subjects (*n* = 984).	The current study attempts to answer the as yet open question of coherence differences between children with ASD and neuro-typical healthy controls. EEG coherence data was evaluated in a large sample of children with ASD and compared to a large neurotypical, medically healthy, normal, age-comparable control group.	A stable pattern of EEG spectral coherence was found to distinguish children with ASD from neurotypical controls.
